# Comprehensive investigations of cerebral hemodynamic responses in CSVD patients with mental disorders: a pilot study

**DOI:** 10.3389/fpsyt.2023.1229436

**Published:** 2023-09-19

**Authors:** Dan Wen, Yong Xu

**Affiliations:** ^1^Department of Psychiatry, First Hospital of Shanxi Medical University, Taiyuan, Shanxi, China; ^2^Shanxi Key Laboratory of Artificial Intelligence Assisted Diagnosis and Treatment for Mental Disorder, First Hospital of Shanxi Medical University, Taiyuan, Shanxi, China; ^3^Department of Psychiatry, Shanxi Medical University, Taiyuan, Shanxi, China

**Keywords:** cerebral small vessel disease (CSVD), mental disorders, blood oxygenation, cerebral blood flow, functional near-infrared diffuse optical spectroscopy (fNIRS), diffuse correlation spectroscopy (DCS), vascular responses

## Abstract

Although a portion of patients with cerebral small vessel disease (CSVD) present mental disorders, there is currently a lack of appropriate technologies to evaluate brain functions that are relevant to neurovascular coupling. Furthermore, there are no established objective criteria for diagnosing and distinguishing CSVD-induced mental disorders and psychiatric diseases. In this study, we report the first comprehensive investigation of the cerebral hemodynamics of CSVD patients who also presented with mental disorders. Two CSVD patients with similar magnetic resonance imaging (MRI) outcomes but with non-identical mental symptoms participated in this study. The patients were instructed to perform the verbal fluency task (VFT), high-level cognition task (HCT), as well as voluntary breath holding (VBH). A functional near-infrared spectroscopy (fNIRS) was used to measure the cerebral oxygenation responses. Additionally, a diffuse correlation spectroscopy (DCS) was used to measure the cerebral blood flow (CBF) responses. Both technologies were also applied to a healthy subject for comparison. The fNIRS results showed that both CSVD patients presented abnormal cerebral oxygenation responses during the VFT, HCT, and VBH tasks. Moreover, the patient with cognition impairment showed fluctuations in CBF during these tasks. In contrast, the patient without cognition impairment mostly presented typical CBF responses during the tasks, which was consistent with the healthy subject. The cognitive impairment in CSVD patients may be due to the decoupling of the neurons from the cerebrovascular, subsequently affecting the autoregulation capacity. The results of the fNIRS and DCS combined provide a comprehensive evaluation of the neurovascular coupling and, hence, offer great potential in diagnosing cerebrovascular or psychiatric diseases.

## 1. Introduction

Cerebral small vessel disease (CSVD) is a common chronic vascular disease that affects millions of people worldwide, particularly the population over 60 years old (1, 2). It is also one of the primary causes of ischemic stroke and dementia due to the abnormalities in arterioles, small veins, and capillaries. In addition, CSVD is found relevant to psychiatric diseases, which account for the largest portion of the global burden of diseases (3). Among psychiatric diseases, depressive disorder is the most common and widespread, accompanied by other psychological and cognitive symptoms (4). Mental disorders, including anxiety and depression (A&D), cognitive decline, and even schizophrenia (5, 6), are also found in a portion of CSVD patients (1, 2). Generally, the prevalence of mental disorders in CSVD patients is inconsistent across the literature, ranging from 10.1 to 34.5% (7–9).

Although relevant to each other, the diagnostic criteria for CSVD and psychiatric diseases are quite different. Magnetic resonance imaging (MRI) plays an important role in diagnosing CSVD. Most abnormalities in the cortex, white matter, and deep structures of the brain, such as small subcortical infarcts, white matter hyperintensities, lacunes, cerebral microbleeds, enlarged perivascular spaces, and cerebral atrophy, can be probed by MRI (10, 11). Nevertheless, a few neurodegenerative diseases have no obvious injuries in small or micro-vessels and are beyond the detecting capability of MRI.

Some reports (8, 12, 13) attribute mental disorders (such as apathy, depression, anxiety, cognitive or motor impairment) in CSVD patients to white matter hyperintensities, lacunar infarcts, or brain atrophy, although other studies do not support this hypothesis (7, 14). Multiple factors, including neuron or vascular deficits, are said to contribute to the causes of mental disorders in CSVD patients. Nevertheless, extensive studies on neuron or vascular functions in CSVD populations, especially those with mental disorders, are rare.

In contrast to CSVD, the diagnosis of psychiatric diseases relies mainly on the subjective questionnaire or screening charts, defined in the fifth edition of the Diagnostic and Statistical Manual of Mental Disorders (DSM-V) and the 10th revision of the International Classification of Diseases (ICD-10) (15, 16).

Currently, there is a gap between CSVD and psychiatric diseases in diagnostic screening tools, though both diseases share similar subjective mental symptoms. Recent advances in neuroscience demonstrate that CSVD may cause damage to neurovascular units, which was found in a few of the population with psychiatric diseases.

The neurovascular unit (NVU), defined to specify the relationship between the brain cells and cerebral vascular, was recently found to be associated with many neurological and vascular diseases, including CSVD and psychiatric diseases (17). It has been reported that an injury to the NVU would cause abnormalities in neural signal transmission, vascular dilation or constriction, or the decoupling between the neurons and the vascular. These functional abnormalities are often not detectable using morphological technologies such as the MRI. On the other hand, though the early stages of CSVD may not alter brain structure, it presents behavior or mental abnormalities similar to those of psychiatric patients. Hence, there are clinical demands for developing brain functional modalities to probe the early stage of CSVD or distinguish between CSVD and psychiatric diseases.

Currently, the available technologies for non-invasive detection of cerebral functional state include functional magnetic resonance imaging (fMRI), electroencephalography (EEG), and functional near-infrared diffuse optical spectroscopy (fNIRS) (18). The fMRI provides the brain's tomographic images of blood oxygenation level dependence (BOLD), which reflect the neuron activities at high spatial resolution (13, 14). Nevertheless, fMRI is expensive and needs heavy instruments with less mobility; thus, it is not ideal for dynamic measurements (14). The EEG is able to capture electrical waves, primarily from the brain cortex, by placing the electrodes over the scalp (19). It is an easy-to-use and high-temporal resolution neurological technology that has been widely used to evaluate brain functions of healthy individuals and those with psychiatric diseases (19, 20). Despite its merits, EEG is subject to a low signal-to-noise ratio and is susceptible to motion artifacts such as blinking and muscular contractions (21).

fNIRS is also a non-invasive technology for measuring the concentrations of oxygenated hemoglobin ([HbO_2_]), deoxygenated hemoglobin ([Hb]), as well as oxygen saturation in biological tissues, primarily the brain cortex, by using the light in near-infrared range and the relevant algorithm such as the modified Beer–Lambert law (18, 22). Compared to fMRI, fNIRS detection is inexpensive, portable, and enables fast measurements. Although the temporal resolution is not as high as that of EEG, fNIRS permits direct assessment of brain oxygenation ([HbO_2_], [Hb], or oxygen saturation) that is closely relevant to tissue metabolism. Recently, fNIRS has been adopted to assess CSVD (23) and a variety of psychiatric diseases, including MDD, A&D, and schizophrenia (24, 25).

Despite its merits, the oxygenation measured by fNIRS only reflects the valance between the oxygen supply and consumption, and it does not directly assess vascular function. Recently, a relatively new NIRS, namely, diffuse correlation spectroscopy (DCS), has been developed for assessing blood flow at the microvasculature level (26–31). Unlike conventional fNIRS that use static light intensity to calculate tissue oxygenation, DCS utilizes the fast fluctuations of the light electric field to quantify the movement of red blood cells (RBCs); thus, it is known as “dynamic NIRS.” The quantification of RBC movements is generally considered a direct approach to measuring the microvasculature blood flow. The measurement of tissue blood flow by DCS has been validated by other flow modalities, including power Doppler ultrasound, laser Doppler, fluorescent microsphere blood flow measurement, and perfusion magnetic resonance imaging (ASL-MRI) (32). Compared to those technologies, DCS offers several advantages, such as non-invasiveness, portability, high temporal resolution (up to several milliseconds), and relatively large penetration depth (up to several centimeters) (32–37). These advantages promote the rapid adoption of DCS in a variety of physiological or clinical studies.

It is well-known that CSVD affects the vasodilation or constriction functions of microvessels, causing deficiencies in tissue hemodynamics. However, these vascular functions can only be assessed through dynamic hemodynamic monitoring rather than static or one-time morphological measurements such as MRI. Although transcranial Doppler (TCD) is routinely used in clinics, it can only assess the blood flow at large and major vessels (e.g., middle cerebral artery). The functional evaluations of CSVD, which affects the cerebral hemodynamics at the arteriole, venule, or capillary level, still remain challenging.

This is a pilot study that investigates the CBF and cerebral oxygenation responses in CSVD patients who also presented mental disorders. A variety of physiological protocols, including verbal fluency task (VFT), high-level cognition task (HCT), as well as voluntary breath holding (VBH), were applied to the patients. These protocols stimulate neuron activities, promote oxygen metabolism, or activate vasoconstriction, all of which were evaluated by the fNIRS and DCS technologies.

To the best of our knowledge, this study is the first comprehensive evaluation of cerebral hemodynamics (oxygenation and CBF) and microvasculature features in CSVD patients with mental disorders. With the optical sensors placed on the frontal head, the cerebral hemodynamics were examined and compared with those of the healthy subject. The cerebral hemodynamic responses derived from optical signals were then correlated with the clinical symptoms of patients to identify the neurological deficits and vascular injuries as well as explain the etiology of these mental disorders.

## 2. Method

### 2.1. Subjects and protocols

A male and a female patient were recruited from the Department of Neurology, First Hospital of Shanxi Medical University, to participate in this study. Both were diagnosed with CSVD, according to the MRI images and clinical features.

The male subject, aged 59, was found to have multiple small infarcts in MRI images scattered over the parietal lobes, prefrontal lobes, and temporal lobes, which indicated CSVD. His major mental symptoms were low mood, decreased pleasantness, and indifference to the surroundings, with urinary incontinence. His responses to instructions were slower than normal individuals, and he had difficulty recognizing, walking, and undertaking other daily activities. The male patient sometimes expressed anger, upset, and delusion of persecution. The female patient, aged 69, was also identified as CSVD by MRI images, as evidenced by the comprehensive distribution of small pieces of infarcts over the parietal lobes and prefrontal lobes. Her major mental symptoms were headaches and dizziness, accompanied by sleeplessness and minor depression.

Both patients had a history of hypertension and were on medication to manage their blood pressure levels. The male patient also had a history of diabetes and hyperlipemia, which were under control by taking the corresponding drugs. The MRI and TCD of the two patients revealed no other abnormalities in the brain structure and functions (e.g., hippocampus or carotid artery flow velocity). In general, the two patients had similar clinical diagnoses according to the current standards (e.g., MRI, TCD, and blood pressure) but with different mental symptoms. The primary motivation of this study was to explore these differences. A healthy male subject, aged 65, was also recruited to participate in this study. The healthy subject was not found to have CSVD or mental disorder, according to the clinical criteria; thus, he was selected as a comparison.

The quantitative Mini Mental State Examination (MMSE), Hamilton Anxiety Rating Scale (HAMA), and Hamilton Depression Rating Scale (HAMD) were administered to the two patients and the healthy subject. The male patient received scores of 22 (MMSE), 16 (HAMA), and 16 (HAMD), demonstrating his cognition deficits, anxiety, and depression. In contrast, the female patient and the healthy subject received a MMSE score higher than 28 and a HAMA score lower than 7. For the HAMD, the healthy subject had a score of 5, while the female patient had a higher score of 9, indicating her minor depression. All the subjects had an education level of high school or above and could understand the instructions. They were all native speakers of Mandarin Chinese and were able to readily cooperate with this study. The study was approved by the Ethics Committee of the First Hospital of Shanxi Medical University. Each subject was informed of the study details and signed a consent form.

### 2.2. Physiological protocols

#### 2.2.1. Verbal fluency task

VFT is a widely used protocol that stimulates neuron excitability (38, 39). We used it in this study for a total of 160 s. This protocol starts with a baseline measurement for 30 s, during which time the subjects sit in a chair and keep their head still. In the next 60-s task period, the subjects are asked to construct as many phrases as possible with the simple words given by the instructor, such as white, small, earth, or rice. At the end of the phrase construction, the subjects are asked to remain still for a recovery period of 70 s.

#### 2.2.2. High-level cognition task

The HCT is similar to VFT, except during the task period. Instead of constructing phrases with simple words, the subjects are instructed to imagine themselves in a particular environment (e.g., forest, grassland, or island) where they need to survive (40). The subjects are then asked a few questions, such as how to find food, how to make tools, and how to defend themselves against threats. Compared to the VFT, the HCT stimulates neuron excitability at a higher intensity.

#### 2.2.3. Voluntary breath holding

The VBH is similar to VFT and HCT, except during the task period. During the VBH, the subjects are instructed to hold their breath as long as possible. The instructor monitors each subject's status and marks the events related to VBH, such as the start and end of breathing. Unlike the VFT and HCT, which initially stimulate neuron excitability, the VBH assesses the complicated mechanism of hypoxia sensing, NVU coupling, as well as vasodilation or constrictions (41).

Of the above three protocols, VFT and HCT are considered active tasks for assessing advanced cognition (33, 34). The VFT primarily assesses the ability to pick up words and extract the related concepts. The HCT further assesses the ability in judgment, conception, and reasoning. The neuron activities and oxygen kinetics are elicited during the VFT and HCT protocols. In contrast, the VBH is considered a passive task to induce transient cerebral ischemia, during which the vascular adaptations (e.g., hypoxia sensing, vasoconstriction) are challenged (41).

Each subject was instructed to perform the three physiological protocols (VFT, HCT, and VBH) sequentially, at intervals of at least 20 min, to avoid interference among the protocols. To minimize variability, these protocols were repeated three times on different days. The average outcomes from these protocols are presented in the Results Section.

### 2.3. Optical measurements

#### 2.3.1. fNIRS for cerebral oxygenation measurement

A 52-channel functional fNIRS device (ETG-4400, Hitachi Medical Limited Inc., Japan) was used for assessing cerebral oxygenation (42). Eleven channels were placed on the prefrontal lobe, and 20 channels on the right and left temporal lobes. Each channel was comprised of one pair of source and detector. Multiple-mode fibers were used for the source and detector.

This sensor arrangement permitted the measurement of [HbO_2_] and [Hb] changes in the dorsolateral prefrontal cortex and right and left temporal cortex (43). According to the principle of fNIRS, the photons emitted from the light source fiber enter the brain cortex via the scalp and skull. Within the tissue, a small portion of photons were scattered multiple times via a “banana shape” trajectory and collected by the detector fiber. Then, the hemodynamic changes were calculated according to the modified Beer-Lambert law (MBLL), a mathematical formula used to calculate the changes in [HbO_2_] and [Hb] from the intensity measurements at two wavelengths (18, 22), with differential pathlength factor (DPF) being set at 5. Two wavelengths (695 and 830 nm) of infrared light were used. The distance between the source and detector was set at 3.0 cm, and the sampling time was set to 0.1 s, from which the activation response of the brain cortex to the stimulus task could be probed.

#### 2.3.2. DCS for cerebral blood flow measurement

The DCS theory and instrumentation can be found in previous literature (33, 35). Briefly, the DCS blood flowmetry device consists of a near-infrared laser at long-coherence length (>5 m), four single-photon-counting photodiode modules, a four-channel digital correlator, and a digital I/O board. A computer panel is used to control the hardware modules. When an optical probe containing the source and detector fibers is placed on the tissue surface (e.g., the frontal head), the DCS laser launches near-infrared photons into the tissue via the source fiber, and the photons within the tissue are absorbed or scattered several times. Ultimately, a small portion of the escaping photons is collected by the detector fiber that is placed several centimeters away from the source fiber. The escaped photons are then counted by a single photon detector and sent to the digital correlator, wherein the normalized function of light intensity (i.e., photon counts) *g*_2_(τ) is calculated. With Siegert relation, the normalized function of light electric field autocorrelation *g*_1_(τ) is derived. Since the unnormalized light electric field autocorrelation function [i.e., *G*_1_(τ)] satisfies a form of partial differential equation (PDE), the blood flow index (α*D*_*B*_) is extracted by fitting the autocorrelation curves with the analytical solution of the PDE (32, 33), or by the Nth-order linear algorithms (34, 44, 45).

The DCS probes (also containing the source and detector fibers) are placed on the prefrontal lobe and temporal lobes for CBF assessment. The fNIRS and DCS sensor (i.e., source or detector fibers) locations are co-registered with the EEG 10/20 international system, ensuring the proper detection of cerebral hemodynamics in the brain cortex. The optical signals derived from the fNIRS and DCS are averaged over all channels, yielding the mean CBF or oxygenation responses to a specific task (VFT, HCT, or VBH).

During the optical measurements, we carefully monitored the subjects and removed the signals probably caused by motion artifacts. Following the guidelines for fNIRS studies (46), a third-order low-pass zero-phase Butterworth filter was designed and applied to all the cerebral hemodynamic data (i.e., [HbO_2_], [Hb], and CBF) in order to remove environmental noises (e.g., instrument noise and cardiac pulsations). The cutoff frequency of the low-pass filter was set at 0.01 to 0.5 Hz. Additionally, the signal noises were further minimized by applying an approach of moving average with a factor of 5 data points.

## 3. Results

### 3.1. VFT responses

**Figure 1:**
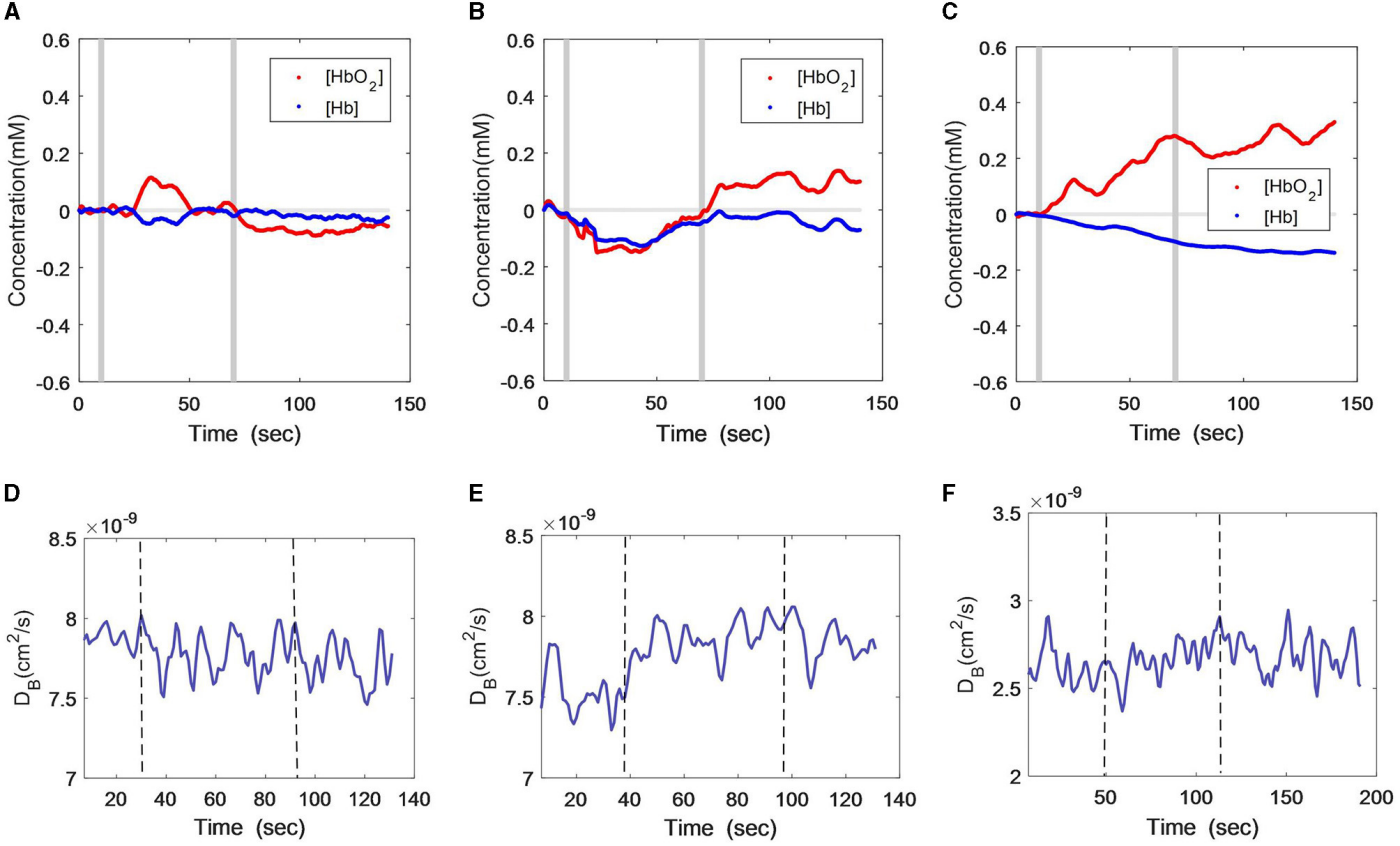
Time-course curves of cerebral oxygenation **(A–C)** and CBF **(D–F)** changes during VFT protocol in the three subjects; **(A)** and **(D)** male patient; **(B)** and **(E)** female patient; **(C)** and **(F)** healthy subject. The gray lines **(A–C)** or dash lines **(D–F)** indicate the start and end of the task.

Figures 1A–C show the [HbO_2_] and [Hb] responses from the three subjects throughout the VFT protocol, wherein the gray lines indicate the start and end of the VFT task. It can be seen that both oxygenation parameters were stable in the male patient when responding to the language test (Figure 1A), and the subject was able to construct the phrases using the given words. While a slight increase in oxygen was found during the task, the increment amplitude was minor. Following the task, there was a slight decline in [HbO_2_], while [Hb] remained at the baseline. The female patient presented a remarkable decline in both [HbO_2_] and [Hb] during the VFT task (Figure 1B), and both variables recovered toward the baseline after the task, especially [HbO_2_], which was even higher than the baseline. In contrast to the two patients, the [HbO_2_] curve of the healthy subject was mostly above zero, with a consistent and large increasing trend (Figure 1C). The [Hb] curve was mostly below zero, with a consistent and large decreasing trend.

The CBF changes during the VFT also differed among the three subjects. As measured by DCS (Figures 1D–F), the male patient did not have remarkable CBF changes but had large fluctuations (Figure 1D). The female subject showed a slight increment in CBF during the VFT task and remained at this level after the task (Figure 1E). In the healthy subject, the CBF remained relatively constant during and after the VFT task (Figure 1F).

### 3.2. HCT responses

**Figure 2:**
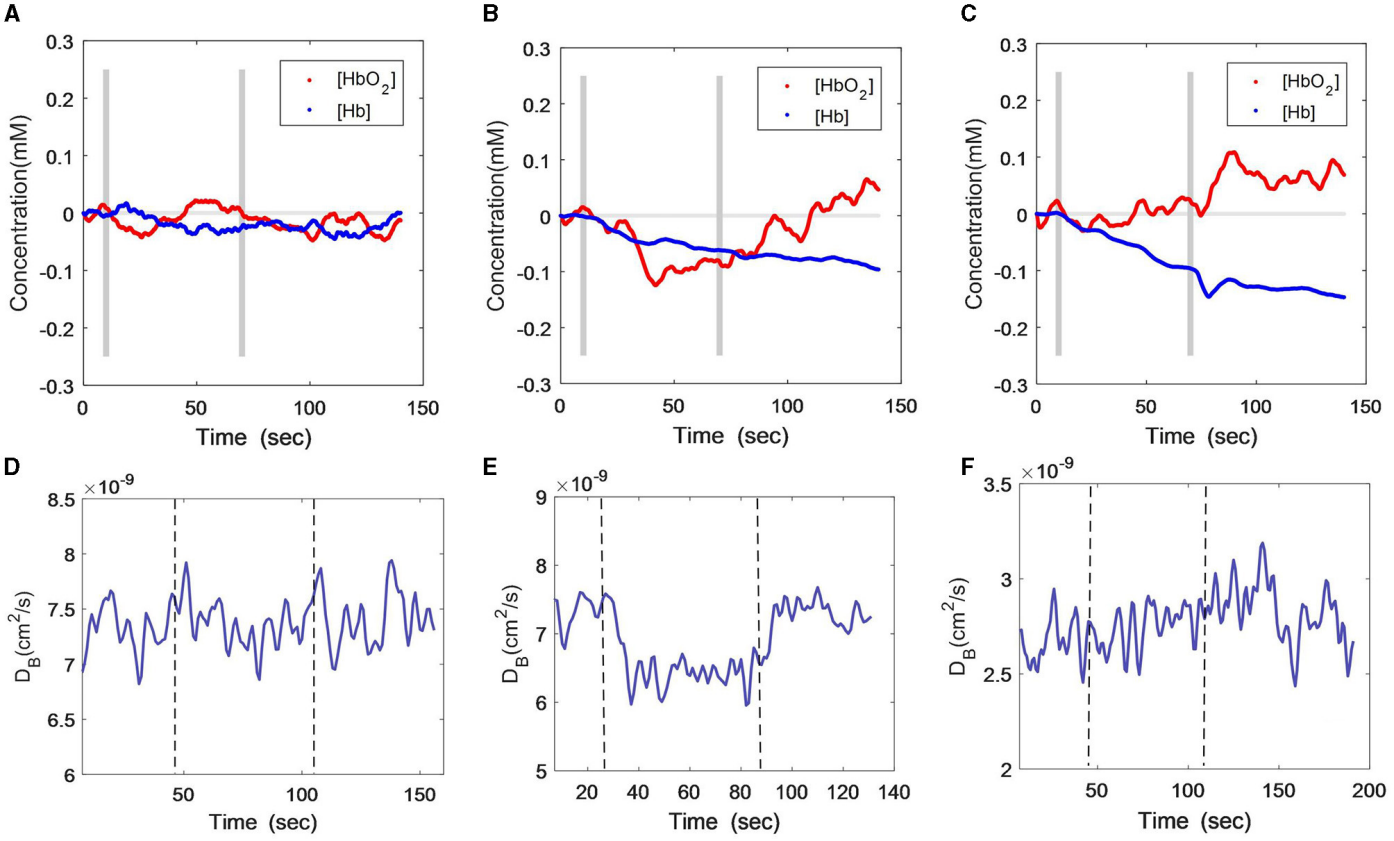
Time-course curves of cerebral oxygenation **(A–C)** and CBF **(D–F)** changes during HCT protocol in the three subjects; **(A)** and **(D)** male patient; **(B)** and **(E)** female patient; **(C)** and **(F)** healthy subject. The gray lines **(A–C)** or dash lines **(D–F)** indicate the start and end of the task.

Figures 2A–C show [HbO_2_] and [Hb] responses of the three subjects throughout HCT. The gray lines indicate the start and end of the cognition stimulus. Similar to the VFT protocol, the male patient did not exhibit obvious changes during or after the HCT period, with only a small variation observed in the oxygenation curve (Figure 2A). The female patient also maintained the VFT protocol features, i.e., both [HbO_2_] and [Hb] declined during HCT, but [HbO_2_] recovered toward the baseline a few seconds later after HCT (Figure 2B). For the healthy subject, there was a [HbO_2_] fluctuation at the start of HCT, after which it continued to increase (Figure 2C). The [Hb] consistently declined, similar to the curve feature of the VFT protocol.

With the DCS measurement (Figures 2D–F), the CBF of the male patient also exhibited large fluctuations during the HCT task (Figure 2D), although a little increment was found toward the end of the task. The female patient's CBF declined during the HCT task and rapidly recovered after the task (Figure 2E), which was different from the VFT stimulus. The healthy subject presented a stable and slightly increasing CBF curve throughout the HCT task (Figure 2F), which was similar to his responses during the VFT task.

### 3.3. VBH responses

**Figure 3:**
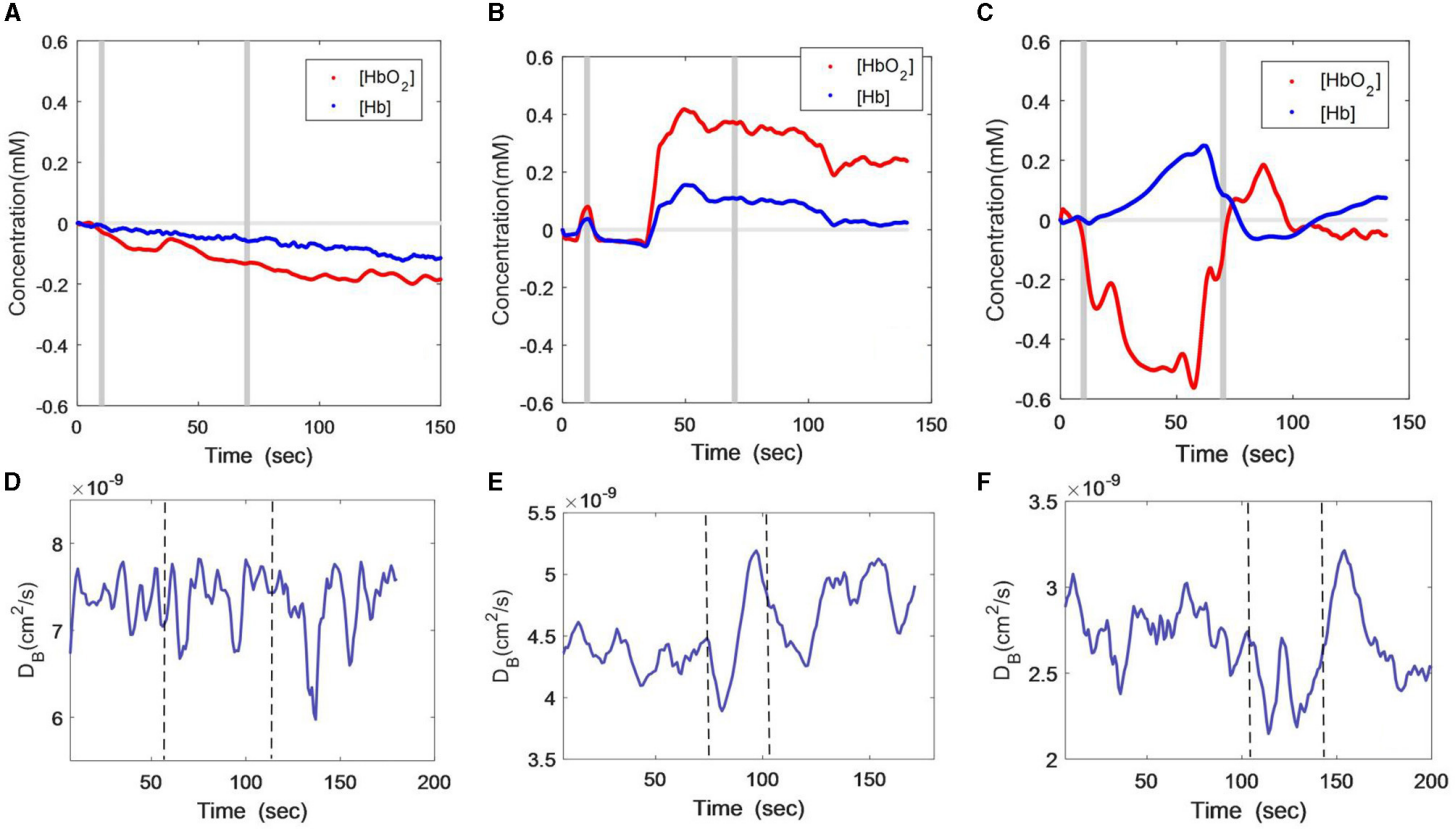
Time-course curves of cerebral oxygenation **(A–C)** and CBF **(D–F)** changes during VBH protocol in the three subjects; **(A)** and **(D)** male patient; **(B)** and **(E)** female patient; **(C)** and **(F)** healthy subject. The gray lines **(A–C)** or dash lines **(D–F)** indicate the start and end of the task.

Figures 3A–C show the [HbO_2_] and [Hb] responses of the three subjects throughout VBH. The gray lines indicate the start and end of the hypoxia stimulus. As illustrated in Figure 3A, the male patient showed a small but consistent decline in both [HbO_2_] and [Hb] during and after VBH. In contrast, the [HbO_2_] and [Hb] of the female remained constant for several seconds. Then, both variables were elevated to the plateau and gradually recovered toward the baseline at the end of the VBH (Figure 3B). The healthy subject exhibited distinct trends, i.e., [HbO_2_] was decreased, while [Hb] was increased during the VBH. Both variables recovered rapidly toward the baseline at the end of the VBF (Figure 3C).

Figures 3D–F show the DCS measurement of the CBF responses to VBH in the three subjects. As with the VFT and HCT protocols, the CBF of the male patient still presented large fluctuations during the VBH task and at the early stages of post-VBH (Figure 3D). The CBF of the female patient was found to rapidly decline to the nadir at the onset of VBH. Then, the CBF started to elevate until the end of VBH. There was a CBF drop toward the end of VBH, after which the CBF recovered toward the baseline (Figure 3E). The CBF of the healthy subject exhibited an inconsistent declining trend during the VBH task. At the end of the VBH, there was a sharp increase in CBF, which then gradually recovered toward the baseline (Figure 3F).

## 4. Discussion

Cerebral small vessel disease is a common brain disease that severely affects the patient's quality of life worldwide. Although most cases of CSVD can be diagnosed by MRI, the degree to which the brain functions are injured by the small vessel deficits still remains unclear. As a consequence, CSVD patients with similar imaging diagnoses would have distinct clinical symptoms or mental disorders. Hence, functional assessment of CSVD is essential for adopting appropriate medical strategy and predicting clinical outcomes, in addition to morphological modalities such as MRI. EEG is a conventional technology to evaluate brain status by detecting the electrical activities of neurons, making it one of the major brain function technologies. However, EEGs are susceptible to electromagnetic noises, and their sensitivity varies across different physiological protocols. The fNIRS technology allows for tissue oxygenation measurement and reflects the oxygen kinetics and metabolic activities. Therefore, it is becoming a popular tool for brain functional assessment. In this study, a new approach, namely, diffuse correlation spectroscopy (DCS), was adopted for the first time to assess the feasibility of cerebral blood flow (CBF) measurement in the CSVD population, in addition to measuring cerebral oxygenation using fNIRS. The cerebral oxygenation measured by fNIRS reflects the balance between oxygen supply and oxygen consumption. Hence, the investigation of cerebral oxygenation could explore the neural activities that involved oxygen metabolism. On the other hand, the CBF measured by DCS reflected the features of vascular vasoconstriction that controlled the blood flow into the tissues. Hence, investigation of CBF could explore the vascular responses and the NVU functions. Therefore, a combination of fNIRS and DCS would provide a comprehensive evaluation of the neuron and vascular deficits in CSVD patients, which would help understand the mechanism behind the development of mental disorders.

The aim of this pilot study was to assess the cerebral hemodynamics, microvascular functions, neuronal excitability, and neurovascular coupling of CSVD patients through a variety of physiological challenges, including VFT, HCT, and VBH. Among those physiological protocols, VFT and HCT primarily elicit neuron excitability with different intensities of cognition as well as the rapid responses of cerebral blood flow, while the VBF challenges the cerebral autoregulation capacity in hypoxia.

A male and a female patient with similar CSVD diagnosis by MRI were selected as the subjects. In addition, the fNIRS and DCS measurements from a healthy subject were also obtained for comparison. The experimental results revealed the neural and vascular characteristics of each subject. According to normal physiological responses, VFT and HCT would induce a large and consistent increment in [HbO_2_] and a decline in [Hb] because the neuron excitability needs an abundant supply of oxygen. This demand triggers the microcirculation to provide more oxygen, usually exceeding what the tissue needs. These features of the fNIRS curves were widely reported in previous studies (22, 39, 40). Due to the autoregulation of the human brain, the CBF tends to remain unchanged during the low (VFT) or moderate (HCT) cognition stimulus. During the VBH, there is insufficient oxygen supply to the tissue because of a breathing pause. As such, [HbO_2_] would be decreased and [Hb] increased, demonstrating typical hypoxia features. Although the small vessels or capillaries could sense the hypoxia via NVU coupling, the tissue tends to adapt to this hypoxia by vasoconstriction. Hence, the CBF would remain constant or have a slight decline at the start of the VBH. When the tissue hypoxia reached the level of tolerance, vasodilation would occur, leading to rapid CBF elevation. At the end of VBH, there would be a hyperemia response characterized by a sharp elevation and a drop in CBF.

Overall, the cerebral oxygenation and CBF responses of the healthy subject matched with all the norm curves mentioned above, except the oxygenation during the HCT protocol. Compared with VFT, the imagination of the subject during the HCT is more self-motive and thus varies across the subjects. This may explain why there were [HbO_2_] fluctuations in our healthy subject at the start of HCT, prior to responding normally to the HCT stimulus.

In contrast to the healthy subject, the male subject presented low amplitudes of oxygenation change, regardless of the tasks—VFT, HCT, or VBH. Additionally, huge fluctuations in CBF were found during all the protocols. These consistent outcomes verified that the male subjects might have deficits in neuron excitability, leading to low oxygen metabolic rates. As a result, [HbO_2_] and [Hb] demonstrated little variation during any of the tasks. The huge fluctuations in CBF might be due to the injuries to the cerebral autoregulation capacity. The deficiency in neurovascular signal transmissions makes vascular vasodilation or vasoconstriction alternate at the vascular inherent frequency instead of persisting.

The [HbO_2_] and [Hb] curves of the female patient were distinctly different from the healthy subject and the male patient. During the VHT, the [HbO_2_] and [Hb] were declined, demonstrating the ischemic phenomenon. This tissue ischemia may be due to the long-term, chronic, insufficient oxygen supply, especially when neuron excitability is activated during the cognition stimulus (VFT and HCT). Although the CBF tends to elevate to improve the microcirculation, there is still a lack of oxygen supply. During VBH, the CBF of the female patient was found to decrease and then increase, which coincided with the oxygenation response, i.e., oxygen remained unchanged for a while and then elevated. These observations indicate that the female patient may have intact neural functions but with damages to the vascular functions, as evidenced by the consistent ischemic phenomenon in all protocols.

The above hemodynamic responses were in agreement with the patient's subjective symptoms. The neural deficits in the male patient, as illustrated by the fNIRS measurement, would be the cause of his mental disorders, inefficient cognition, and urinary incontinence. Additionally, the male patient was also impaired in vascular functions, which was depicted by the huge fluctuations in his CBF measured using DCS. The fNIRS measurements revealed that the female patient had normal oxygen consumption, indicating that she maintained good neuron excitability. As such, the female patient did not present cognition deficits or serious mental disorders. Nevertheless, the female patient might be impaired in vascular functions that led to chronic ischemia, as evidenced by the declined oxygenation during cognition stimulus. Compared to the male patient and the healthy subject, the cerebrovascular of the female patient was more sensitive to any of the stimulus protocols, evidenced by the significant changes (i.e., increased or decreased) in CBF. These observations indicate that the female patient may have a deficiency in cerebral autoregulation capability, which explains why she often experienced headaches and dizziness. Chronic ischemia and hypoxia can also affect neural function, such as insomnia and minor depression.

From the above observations, it is clear that CSVD and psychiatric patients may have different etiologies for their mental disorders. Vascular impairments seen on MRI images of CSVD patients may affect vascular vasoconstrictions or neurovascular coupling, which might be the reason for mental disorders in some CSVD patients. In contrast, there is little evidence of vascular impairments in people with psychiatric diseases, i.e., most psychiatric patients have normal medical images in the brain structure. The possible mechanisms for those patients might be neurological deficits, which cause mental disorders such as anxiety, depression, and cognition declines. Comparisons between a larger number of CSVD patients and psychiatric patients with fNIRS and DCS measurements would help reveal the differences and elucidate the mechanism of mental disorders, which could be the subject of future research.

The above hypothesis on different etiologies for CSVD and psychiatric patients was partially verified in the current study. Specifically, the healthy subject exhibited normal curves of CBF and oxygenation when responding to all three physiological protocols (Figures 1–3), implying intact functions in both neuron excitation and vascular vasoconstriction. The female CSVD patient, with only minor depression, was found to have mostly normal curves in oxygenation response, indicating her healthy status in neuron activities. Her ischemic response in CBF curves (Figure 2E) could be due to vascular impairments, which was confirmed by the MRI images. In contrast, the male CSVD patient was found to have abnormal curves in both cerebral oxygenation and CBF (Figures 1–3), which are consistent with his clinical diagnostic outcomes, i.e., small vessel deficits with comprehensive mental disorders.

The major limitation of this study is the small number of cases, i.e., only two CSVD patients (with and without mental disorders) and a healthy subject were included in the study. As a pilot study, we aimed to evaluate the feasibility of the cerebral hemodynamics data, especially the blood flow, using DCS, for the assessment of neuron or vascular deficits in the CSVD population. We observed the differences between the three subjects in CBF and oxygenation responses, demonstrating the clinical implications of using the cerebral hemodynamic responses to assess neurovascular functions or NVU, which are very relevant to mental health. In the future, an extensive study involving a large number of participants can be conducted to reach a solid conclusion with statistical outcomes.

In summary, this study reports case investigations on CSVD patients with different degrees of mental disorders using fNIRS and the novel DCS. Three physiological protocols, i.e., VFT, HCT, and VBH, were selected as the stimulus tasks to evaluate the cerebral oxygenation and CBF responses. Because of the considerable variability among CSVD patients in terms of disease and medication history, it is difficult to categorize the patients according to clinical standards such as MRI. Hence, instead of reporting the group outcomes, we examined the cerebral hemodynamic responses of each subject, which is more accurate for functional evaluation of individual NVU and cerebral autoregulation capacity as well as for better individualized healthy management. We found that the male and female CSVD patients in our study, although with similar morphological diagnoses, presented different abnormal features in time-course curves of cerebral oxygenation and CBF. These hemodynamic abnormalities indicate their deficiency in neuron activities, neurovascular coupling, as well as vascular dilation or constriction capability. In addition to the case investigations, this is the first study that has combined fNIRS and DCS for a comprehensive assessment of neuron excitability, neurovascular coupling, and vascular functions. The individuals presented unique hemodynamic features that coincided with their subjective symptoms. Further exploration of hemodynamic data could help explain the underlying mechanisms of CSVD and mental disorders, hence assisting in accurate diagnosis of CSVD and psychiatric diseases.

## Data availability statement

The raw data supporting the conclusions of this article will be made available by the authors, without undue reservation.

## Ethics statement

The studies involving humans were approved by the Ethics Committees of the First Hospital of Shanxi Medical University. The studies were conducted in accordance with the local legislation and institutional requirements. The participants provided their written informed consent to participate in this study. Written informed consent was obtained from the individual(s) for the publication of any potentially identifiable images or data included in this article.

## Author contributions

DW and YX conceived of the study design. DW managed the literature searches, collected data, undertook the data analysis under the supervision of YX, and wrote the first draft. YX revised the manuscript. All the authors contributed to and have approved the final manuscript.
